# Diversity of the Genes Implicated in Algerian Patients Affected by Usher Syndrome

**DOI:** 10.1371/journal.pone.0161893

**Published:** 2016-09-01

**Authors:** Samia Abdi, Amel Bahloul, Asma Behlouli, Jean-Pierre Hardelin, Mohamed Makrelouf, Kamel Boudjelida, Malek Louha, Ahmed Cheknene, Rachid Belouni, Yahia Rous, Zahida Merad, Djamel Selmane, Mokhtar Hasbelaoui, Crystel Bonnet, Akila Zenati, Christine Petit

**Affiliations:** 1 Laboratoire de biochimie génétique, Service de biologie - CHU de Bab El Oued, Université d'Alger 1, 16 Alger, Algérie; 2 Laboratoire central de biologie, CHU Frantz Fanon, 09 Blida, Algérie; 3 Faculté de médecine, Université Saad Dahleb, 09 Blida, Algérie; 4 Unité de génétique et physiologie de l’audition, INSERM UMRS1120, Institut Pasteur, 75015, Paris, France; 5 Faculté des sciences biologiques, Université des sciences et de la technologie Houari Boumédiène, 16 Alger, Algérie; 6 Service d’ophtalmologie, CHU Frantz Fanon, 09 Blida, Algérie; 7 Service de biochimie et de biologie moléculaire, Hôpital Armand Trousseau, APHP, 75012, Paris, France; 8 Service d’ORL, CHU Frantz Fanon, 09 Blida, Algérie; 9 Service d’ORL, CHU Bab el Oued, 16 Alger, Algérie; 10 Service d’ORL, CHU Tizi Ouzou, 15 Tizi-Ouzou, Algérie; 11 INSERM UMRS 1120, Institut de la vision, Université Pierre et Marie Curie, 75005, Paris, France; 12 Collège de France, 75005, Paris, France; Institut Curie, FRANCE

## Abstract

Usher syndrome (USH) is an autosomal recessive disorder characterized by a dual sensory impairment affecting hearing and vision. USH is clinically and genetically heterogeneous. Ten different causal genes have been reported. We studied the molecular bases of the disease in 18 unrelated Algerian patients by targeted-exome sequencing, and identified the causal biallelic mutations in all of them: 16 patients carried the mutations at the homozygous state and 2 at the compound heterozygous state. Nine of the 17 different mutations detected in *MYO7A* (1 of 5 mutations), *CDH23* (4 of 7 mutations), *PCDH15* (1 mutation), *USH1C* (1 mutation), *USH1G* (1 mutation), and *USH2A* (1 of 2 mutations), had not been previously reported. The deleterious consequences of a missense mutation of *CDH23* (p.Asp1501Asn) and the in-frame single codon deletion in *USH1G* (p.Ala397del) on the corresponding proteins were predicted from the solved 3D-structures of extracellular cadherin (EC) domains of cadherin-23 and the sterile alpha motif (SAM) domain of USH1G/sans, respectively. In addition, we were able to show that the *USH1G* mutation is likely to affect the binding interface between the SAM domain and USH1C/harmonin. This should spur the use of 3D-structures, not only of isolated protein domains, but also of protein-protein interaction interfaces, to predict the functional impact of mutations detected in the USH genes.

## Introduction

Usher syndrome (USH, MIM 276900, MIM 276905, MIM 605472) is an autosomal recessive disorder that associates sensorineural hearing impairment with retinitis pigmentosa, and in some cases vestibular dysfunction. USH accounts for more than a half of the cases of inherited deaf-blindness, and its prevalence has been estimated at between 1/6000 and 1/25000 [1–4]. USH is clinically and genetically heterogeneous [5, 6]. Three clinical subtypes can be distinguished. USH of type 1 (USH1) is the most severe form, characterized by congenital severe to profound deafness, vestibular dysfunction, and prepubertal onset of the visual loss [2, 6, 7]. USH1 accounts for 30%-40% of USH cases in the European population [2, 4]. Six causal genes have been reported: *MYO7A* [8], *PCDH15* [9], *CDH23* [10, 11], *USH1C* [12, 13], *USH1G* [14], and *CIB2* [15], encoding the actin-based motor protein myosin VIIa (USH1B), the transmembrane proteins protocadherin-15 (USH1F) and cadherin-23 (USH1D), the submembrane scaffold proteins harmonin (USH1C) and sans (USH1G), and the calcium-integrin-binding protein CIB2 (USH1J), respectively. USH of type 2 (USH2) is more frequent, but usually less severe than USH1 as it associates congenital, moderate to severe hearing impairment with visual loss beginning in the first or second decade of life, without vestibular dysfunction. Three causal genes have been identified: *USH2A* [16], *ADGRV1* [17], and *WHRN/DFNB31* [18], encoding the large transmembrane proteins usherin (USH2A) and adhesion G protein-coupled receptor V1 (USH2C), and the submembrane scaffold protein whirlin (USH2D), respectively. Finally, USH of type 3 (USH3) is characterized by progressive hearing loss, variable age of onset of the visual loss, and variable vestibular dysfunction. Only one USH3 gene, *CLRN1*, encoding the transmembrane protein clarin-1, has been reported [19]. USH1 proteins have been shown to interact and to form a molecular complex [20]. USH2 proteins also form a molecular complex [21, 22].

The spectrum of genes and mutations involved in Algerian USH patients is still poorly defined because only a few Algerian patients have been studied so far [23, 24]. We analyzed the molecular bases of USH in 18 unrelated Algerian patients, 16 of them being affected by USH1 and the other two by USH2, by targeted-exome sequencing of the ten identified USH genes.

## Patients and Methods

### Ethics statement

This study was approved by the local ethics committee of the CHU Bab El-Oued, in accordance with the "Loi sanitaire n° 85–05 du 16 février 1985" applicable in Algeria. This study was conducted according to the principles of the declaration of Helsinki. Patients were anonymized, and the corresponding code was conserved in a confidential file. Written consent for genetic testing was obtained from the adult participants and from the parents of minor participants.

### Patients

Eighteen unrelated Algerian USH patients (age ranging from 19 months to 54 years old) from the ENT departments of CHU Blida Hospital (15 patients), CHU Bab El Oued in Algiers (one patient), and CHU Tizi Ouzou (2 patients) were included in this study, together with their clinically unaffected parents. Most patients (14 out of 18) were born to parents known to be consanguineous. Clinical history was taken for each patient. The patients underwent audiograms, ocular fundus autofluorescence imaging, and electroretinogram. All patients had bilateral, moderate to profound deafness, and the 12 patients aged at least five years also had reduced visual field and night vision. All USH1 patients had delayed onset of independent walking (> 18 months) and vestibular dysfunction. The parents of all patients had normal hearing and no retinal degeneration on fundus examination.

### Targeted-exome sequencing

Genomic DNA was extracted from peripheral blood using standard procedures. Targeted-exome sequencing and bioinformatics analysis were performed as previously described [25]. To amplify and sequence all coding exons of the 10 USH genes identified so far (i.e., a total of 366 exons), specific primers were designed using Primer3 (http://bioinfo.ut.ee/primer3-0.4.0/); the sequences of these oligonucleotides are listed in S1 Table. Sequences were run on ABI 3730 DNA analyzer, and compared with Genbank reference sequences (http://www.ncbi.nlm.nih.gov/genbank/) using ABI Prism Seqscape 2.1.

The segregation of the mutations identified in the patients was studied by Sanger sequencing of the corresponding DNA fragments in the parents and other relatives, whenever available. The Sorting Intolerant from Tolerant (SIFT), Mutation Taster, and PolyPhen-2 software programs were used to predict the impact of all amino acid substitutions on the protein structure and function. The NNSplice, ESEfinder, Max Ent Scan, Gene Splicer, and Human Splicing Finder programs were used to predict abnormal splice sites.

### Reference sequences for mutation nomenclature and exon numbering

In this article, the nomenclature of all sequence variants and exon numbering refer to the following genomic and cDNA reference sequences (NG_ and NM_ NCBI accession numbers, respectively): *MYO7A* [NG_009086.1, NM_000260.3]; *USH1C* [NG_011883.1, NM_153676.3]; *CDH23* [NG_008835.1, NM_022124.5]; *PCDH15* [NG_009191.1, NM_033056.3]; *USH1G* [NG_007882.1, NM_173477.4]; *USH2A* [NG_009497.1, NM_206933.2].

All the mutations identified were deposited in the Leiden Open Variation Database (http://www.lovd.nl/3.0/home).

### Mutation modeling

The structural consequences of the two novel missense mutations in *CDH23* and of the in-frame single codon deletion in *USH1G* were analyzed on the 3D-structure models of the cadherin-23 EC1-EC2 & protocadherin-15 EC1-EC2 (PDB 4APX) and sans SAM-PBM & harmonin Nter-PDZ1 (PDB 3K1R) molecular complexes, respectively. The PyMOL software program was used for cartoon representations.

## Results and Discussion

**Fig 1:**
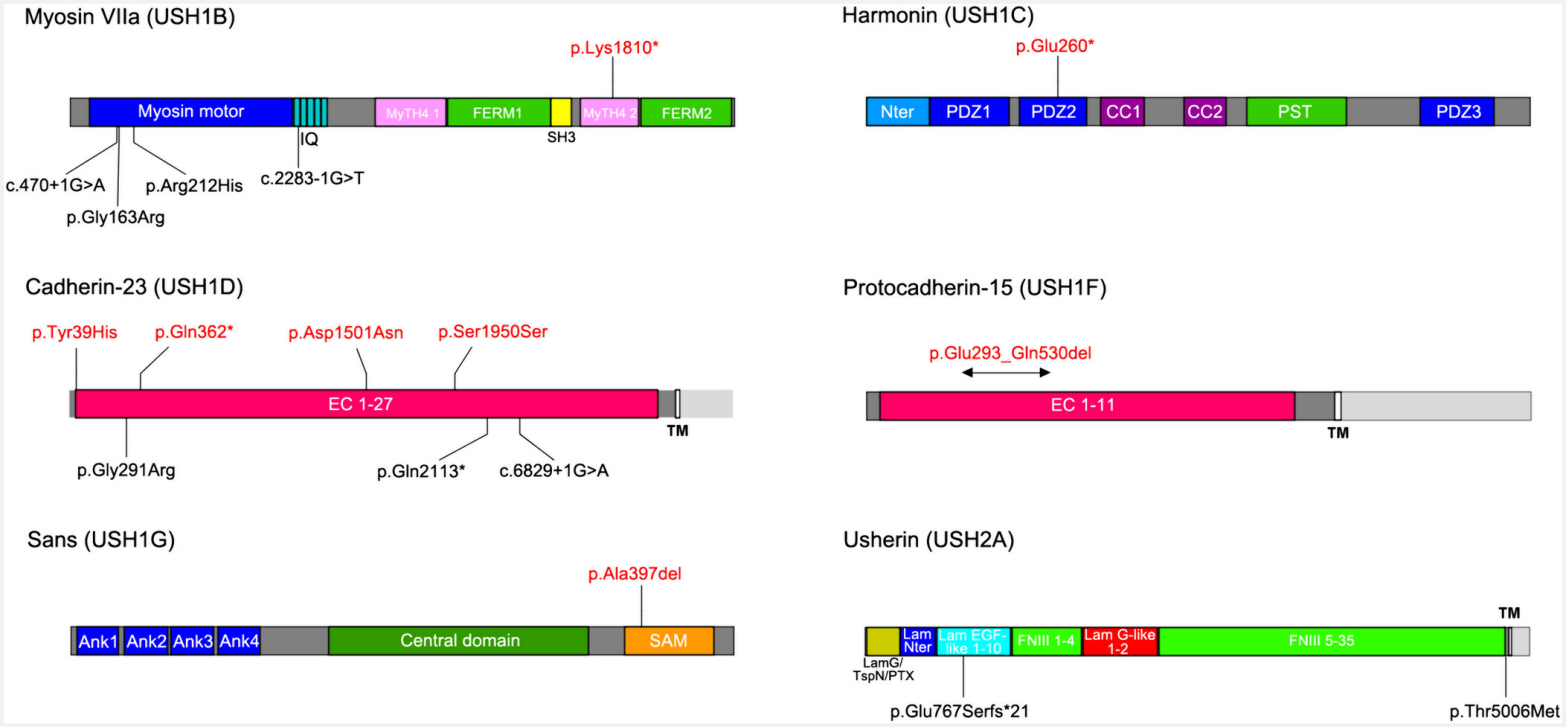
Schematic representation of myosin-VIIa (USH1B), harmonin (USH1C), cadherin-23 (USH1D), protocadherin-15 (USH1F), sans (USH1G), and usherin (USH2A). For each protein, the longest isoform is shown. The novel and the previously reported causal mutations in Algerian USH patients are indicated in red and in black, respectively. Abbreviations: *IQ*, isoleucine-glutamine motifs; *MyTH4*, myosin tail homology 4 domain; *FERM*, band 4.1-ezrin-radixin-moesin domain; *SH3*, src homology 3 domain; *Nter*, N-terminal domain; *PDZ*, PSD95-discs large-ZO1 domain; *CC*, coiled coil domain; *PST*, proline-serine-threonine-rich region; *EC*, extracellular cadherin domain; *TM*, transmembrane domain; *Ank*, ankyrin domain; *SAM*, sterile alpha motif domain; *LamG/TspN/PTX*, N-terminal thrombospondin/pentaxin/laminin G-like domain; *Lam Nter*, laminin N-terminal domain; *Lam EGF-like*, laminin-type EGF-like domain; *Lam G-like*, laminin G-like domain; *FNIII*, fibronectin type III domain.

In a cohort of 180 Algerian deaf patients, we recruited 18 unrelated USH patients (16 USH1 and 2 USH2 patients) on the basis of associated vestibular dysfunction (including delayed onset of independent walking in all 16 USH1 patients) and/or reduced night vision. The presence of a retinal dysfunction was confirmed by electroretinogram analysis. By targeted-exome sequencing of the known USH genes, we found biallelic mutations in all the patients. Sixteen patients carried mutations at the homozygous state, and two patients at the compound heterozygous state. A total of 17 different pathogenic or presumably pathogenic mutations were identified in five USH1 genes (*MYO7A*, *CDH23*, *PCDH15*, *USH1C*, and *USH1G*) and in *USH2A*: 4 nonsense, 1 frameshift, 3 splice-site, and 6 missense mutations, a synonymous mutation predicted to create a splice acceptor site, an in-frame deletion of one codon, and a large intragenic deletion (Table 1, Fig 1, and S1 Fig).

**Table 1:** List of the mutations found in USH genes.

Patient	Age (years)	Gene	Allele 1	Allele 2
ALG01U06S310	8	*MYO7A*	c.5428A>T (p.Lys1810*)	c.5428A>T (p.Lys1810*)
ALG01U03S170	14	*MYO7A*	c.635G>A (p.Arg212His)[26]	c.635G>A (p.Arg212His)[26]
ALG01U0576051	4	*MYO7A*	c.2283-1G>T[27]	c.2283-1G>T[27]
ALG01U04A67100	18	*MYO7A*	c.2283-1G>T[27]	c.2283-1G>T[27]
ALG01U0274761	3	*MYO7A*	c.487G>A (p.Gly163Arg) [27]	c.2283-1G>T[27]
ALG01U0194950	10	*MYO7A*	c.470+1G>A[29]	c.470+1G>A[29]
ALG01U0774920	5	*USH1C*	c.778G>T (p.Glu260*)	c.778G>T (p.Glu260*)
ALG01U0892201	3	*USH1C*	c.778G>T (p.Glu260*)	c.778G>T (p.Glu260*)
ALG01U0974821	3	*CDH23*	**c.115T>C (p.Tyr39His)**	**c.115T>C (p.Tyr39His)**
ALG01U1275791	10	*CDH23*	**c.5850T>A (p.Ser1950Ser)**	**c.5850T>A (p.Ser1950Ser)**
ALG01U1476321	< 2 (19 months)	*CDH23*	c.6829+1G>A[31]	c.6829+1G>A[31]
ALG01U1376351	54	*CDH23*	c.6337C>T (p.Gln2113*)[30]	c.6337C>T (p.Gln2113*)[30]
ALG01U1076470	6	*CDH23*	c.871G>A (p.Gly291Arg)[32]	c.871G>A (p.Gly291Arg)[32]
ALG01U11A64230	7	*CDH23*	c.1084C>T (p.Gln362*)	**c.4501G>A (p.Asp1501Asn)**
ALG01U1574950	17	*PCDH15*	**c.(876+29089)_(1590+3491)del (p.Glu293_Gln530del)**	**c.(876+29089)_(1590+3491)del (p.Glu293_Gln530del)**
ALG01U1663800	9	USH1G	**c.1188_1190del (p.Ala397del)**	**c.1188_1190del (p.Ala397del)**
ALG01U17SA41	26	*USH2A*	c.2299delG (p.Glu767Serfs*21)[16]	c.2299delG (p.Glu767Serfs*21) [16]
ALG01U1881900	18	*USH2A*	c.15017C>T (p.Thr5006Met)[33]	c.15017C>T (p.Thr5006Met) [33]

Novel mutations are indicated in bold.

Nine mutations have previously been reported (see Table 1). The p.Arg212His and p.Gly163Arg missense mutations in *MYO7A* (see Table 2) have also been found in Belgian, Dutch, and American patients [26] and in Algerian and Turkish patients [27], respectively. The c.2283-1G>T splice site mutation in *MYO7A*, first described in an Algerian patient [27], seems to be frequent in Maghrebian populations [28]. We found this mutation in three Algerian patients, at the homozygous and compound heterozygous states. The other splice site mutation in *MYO7A* (c.470+1G>A) has been reported in Tunisian patients [29]. The p.Gln2113*, c.6829+1G>A, and p.Gly291Arg mutations in *CDH23* (see Table 2) have been found in an American family [30], a French family [31], and a Spanish family [32], respectively. The p.Thr5006Met missense mutation in *USH2A* (see Table 2) has been reported in French and Danish patients [33]. Finally, the c.2299delG ancestral mutation in *USH2A* [16] accounts for 47.5% and 15% of the mutant alleles identified in USH2A patients living in Denmark and in Spain, respectively [34, 35], but, to our knowledge, had not yet been reported in patients from North Africa.

**Table 2:** Predicted pathogenicity of the six missense mutations found in Algerian USH patients.

Gene	*CDH23*			*MYO7A*		*USH2A*
Mutation	Tyr39His	Gly291Arg	Asp1501Asn	Gly163Arg	Arg212His	Thr5006Met
SIFT	Deleterious (0)	Deleterious (0)	Deleterious (0)	Deleterious (0)	Deleterious (0)	Deleterious (0)
Mutation Taster	Disease causing (0.94)	Disease causing (0.94)	Disease causing (0.7)	Disease causing (1)	Disease causing (1)	Disease causing (0.99)
PolyPhen-2	Probably damaging (1)	Probably damaging (1)	Probably damaging (1)	Probably damaging (1)	Probably damaging (1)	Probably damaging (1)

The other eight mutations had not been previously reported. Six were present at the homozygous state (6 USH1 patients), and two were present at the compound heterozygous state in an USH1 patient (see Table 1). These mutations were absent in HapMap, 1000 genomes, Exome Variant Server, and Deafness Variation databases, and in the Liveset database (LSDB) for USH genes, and they were not detected in 150 Algerian control individuals. The two missense mutations are located in *CDH23* exon 3 (p.Tyr39His) and exon 36 (p.Asp1501Asn), encoding the first and 14^th^ extracellular cadherin (EC) domains of cadherin-23, respectively. Both mutations were predicted to be pathogenic by PolyPhen-2, Sorting Intolerant from Tolerant (SIFT), and Mutation Taster (see Table 2).

**Fig 2:**
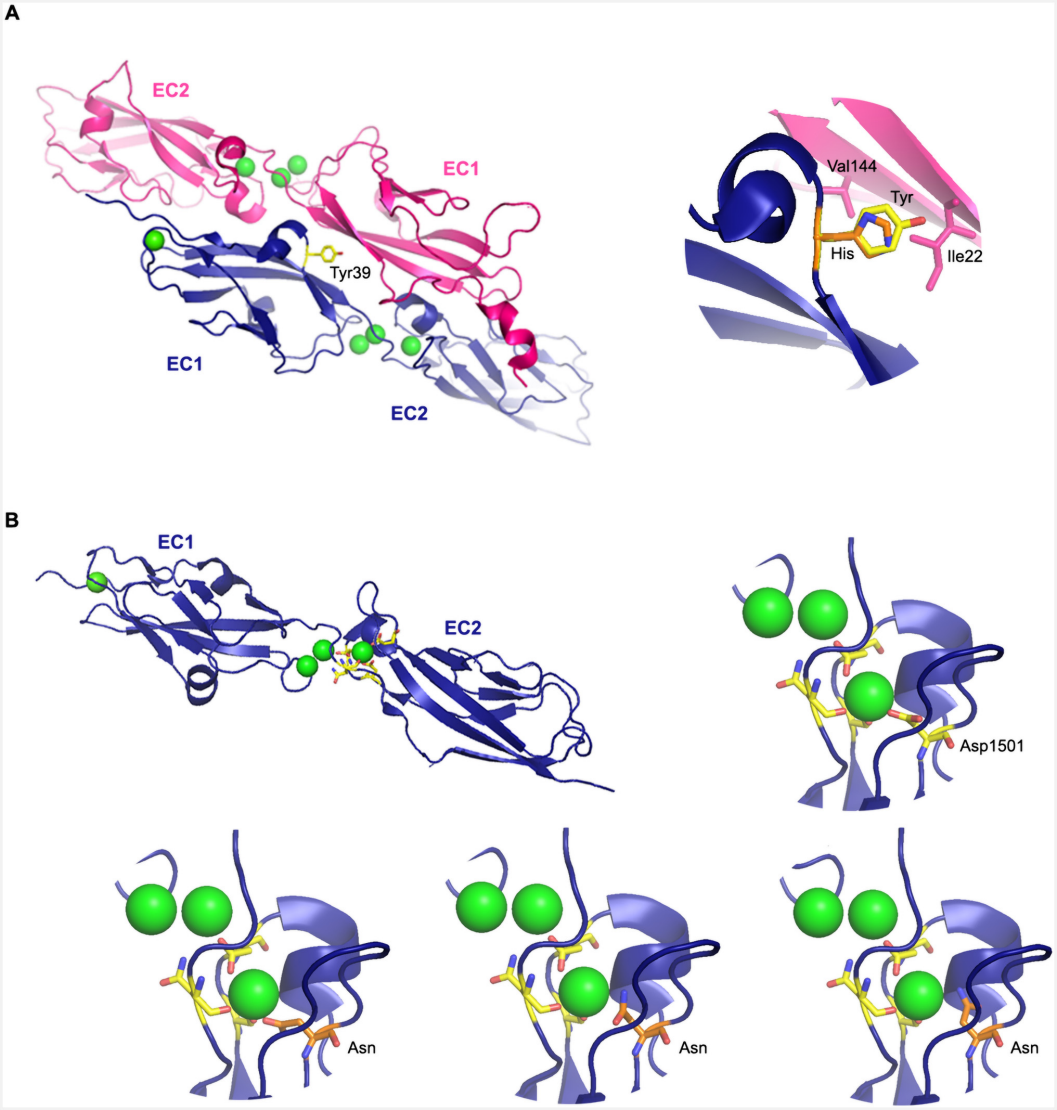
Predicted effect of the p.Tyr39His and p.Asp1501Asn *CDH23* mutations. (A) On the left side, ribbon diagram of protocadherin-15 EC1-EC2 (pink) bound to cadherin-23 EC1-EC2 (blue). Ca^2+^ ions are depicted as green spheres. The Tyr39 residue of cadherin-23 is shown in yellow. On the right side, detailed cartoon of the cadherin23-protocadherin15 interaction interface. The Tyr39 residue engages in van der Waals interactions with Ile22 and Val114 of protocadherin-15, and the substitution of this residue by His (orange) is not predicted to change these interactions. (B) On the left side, ribbon diagram showing the backbone of cadherin-23 EC1-EC2 and the side chains of the amino-acid residues involved at the Ca^2+^-binding interface of the EC1-2 linker. On the right side, detailed cartoon of the corresponding Ca^2+^-binding site in the EC14-15 linker. The Asp1501 residue is shown in yellow. Lower panel: the substitution of this residue by Asn (orange) causes steric clashes or charge incompatibility in all three modeling rotamers, and is therefore predicted to impair Ca^2+^-binding.

The two most amino-terminal EC repeats (EC1+EC2) of cadherin-23 and protocadherin-15 can interact to form an overlapped, antiparallel heterodimer that is believed to be the structural basis of apical fibrous links joining inner ear hair cells’ stereocilia [36]. The Tyr39 residue of cadherin-23 belongs to the loop between the 3_10_ helix and A strand of EC1, and is located at the cadherin23-protocadherin15 interaction interface, but the Tyr39His mutation does not appear to perturb the hydrophobic environment of this surface according to the structural model of the interaction (Fig 2A). However, we cannot exclude the possibility that Tyr39 plays an important role in another mode of cadherin-cadherin interaction that might also be involved in stereocilia links, especially since this residue is located at the N-terminal end of the protein. The other missense mutation of cadherin-23, Asp1501Asn, occurs in the loop between EC14 and EC15. Sequence alignment of the 27 EC repeats of the protein shows the presence of aspartic acid residues at positions equivalent to Asp1501 (S2 Fig), and the solved 3D-structure of EC1-EC2 indicates that the equivalent aspartic acid residue in the EC1-2 linker contributes to the Ca^2+^-binding site (Fig 2B). The substitution of the negatively charged Asp1501 by Asn is predicted to disrupt the corresponding Ca^2+^-binding site (Fig 2B).

**Fig 3:**
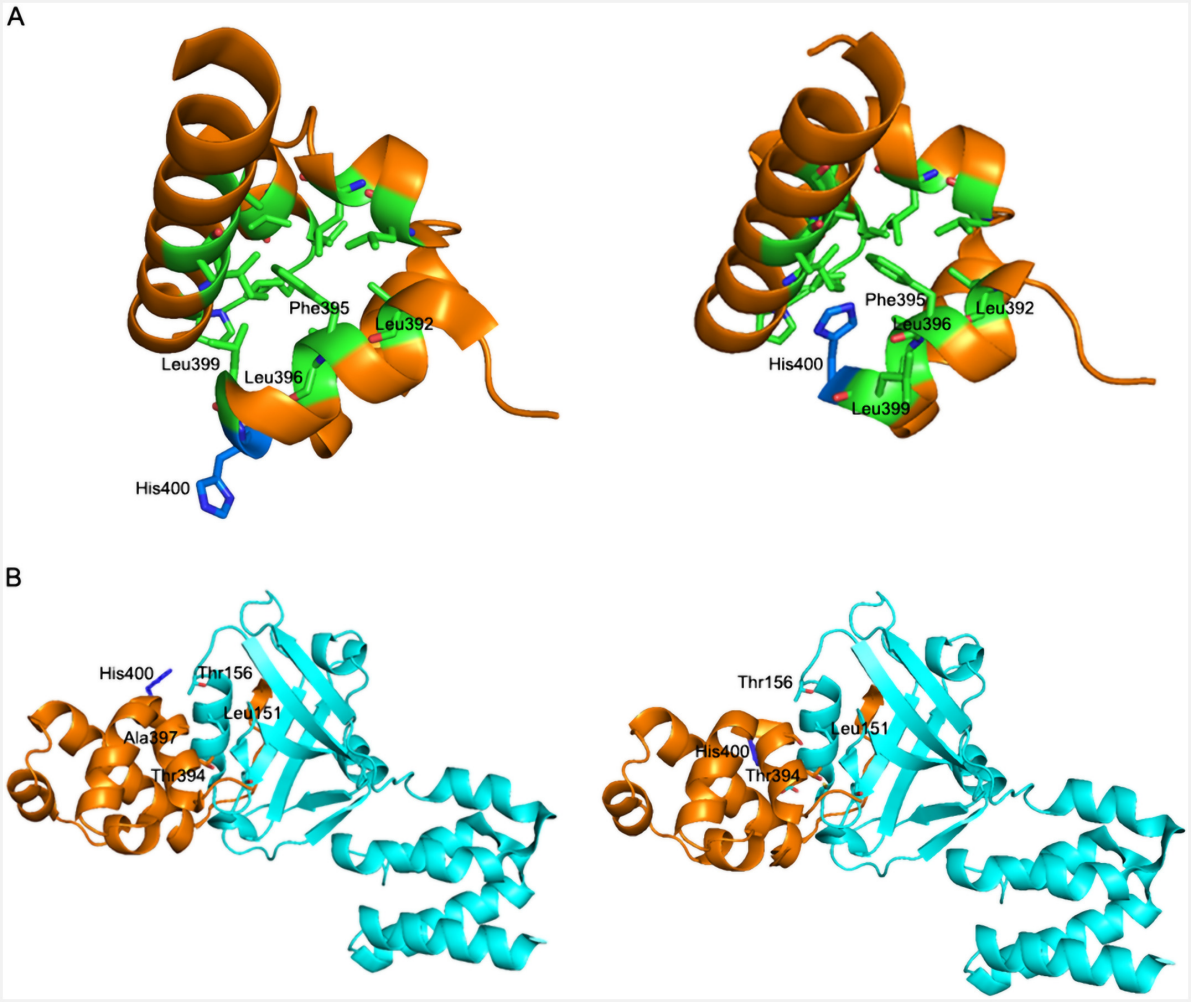
Predicted effect of the p.Ala397del *USH1G* mutation. (A) On the left side, ribbon diagram showing the hydrophobic core of the SAM domain of USH1G/sans. The side chains of the hydrophobic residues Leu392, Phe395, Leu396, and Leu399 and of the polar residue His400 of the αA helix are represented in green and in blue, respectively. On the right side, the ribbon diagram shows the disruption of the hydrophobic core of the SAM domain by the His400 polar residue in the presence of the p.Ala397del mutation. (B) On the left side, ribbon diagram showing the overall structure of the Nter-PDZ1 domain of USH1C/harmonin (cyan) interacting with the SAM domain of USH1G/sans (orange). The αA helix of the SAM domain interacts with the surface groove of the harmonin PDZ1 domain. Note the major involvement of the SAM domain residues Thr394, Ala397, and His400 in this interaction. On the right side, the ribbon diagram shows the abnormal SAM-PDZ1 interaction interface in the presence of the p.Ala397del mutation: the Leu151 and Thr156 residues of the PDZ1 domain cannot interact with the missing Ala397 residue and the His400 residue of the SAM domain, respectively.

The novel in-frame deletion of three nucleotides identified in *USH1G* (p.Ala397del) removes an alanine residue from the first α-helix of the sterile alpha motif (SAM) domain. This helix (αA) is part of the SAM domain hydrophobic core formed by a bundle of four α-helices. Modeling of the mutated SAM domain lacking the Ala397 residue predicts a disruption of the hydrophobic core (Fig 3A). In addition, Ala397 contributes to the interface of interaction between sans (USH1G) and harmonin, the PSD95-discs large-ZO1 (PDZ) domain-containing protein encoded by *USH1C* [14], [37], and the absence of this residue is predicted to affect the docking of the SAM domain αA helix to the surface groove of the harmonin PDZ1 domain (Fig 3B). Incidentally, among the twelve presumably pathogenic mutations previously reported in *USH1G*, there were only two (monoallelic) missense mutations affecting residues located in the SAM domain, p.Leu420Val and p.Arg447Trp, none of which is predicted to affect the binding to harmonin [38, 39].

The three novel nonsense mutations are located in *MYO7A* exon 39 (p.Lys1810*), encoding the second myosin tail homology 4 (MyTH4) domain of myosin VIIa, in *USH1C* exon 10 (p.Glu260*), encoding the PDZ2 domain of harmonin (common to all harmonin classes and subclasses), and in *CDH23* exon 10 (p.Gln362*), encoding the 4^th^ EC domain of cadherin-23. In addition, the novel c.5850T>A (p.Ser1950Ser) synonymous mutation in *CDH23* is predicted to create a splice acceptor site in exon 44 (encoding the 18^th^ EC domain of the protein), which also results in a premature stop codon in the mature transcript. All these mutations are expected to result either in a truncated protein or in no protein at all owing to nonsense mediated mRNA decay [40].

**Fig 4:**
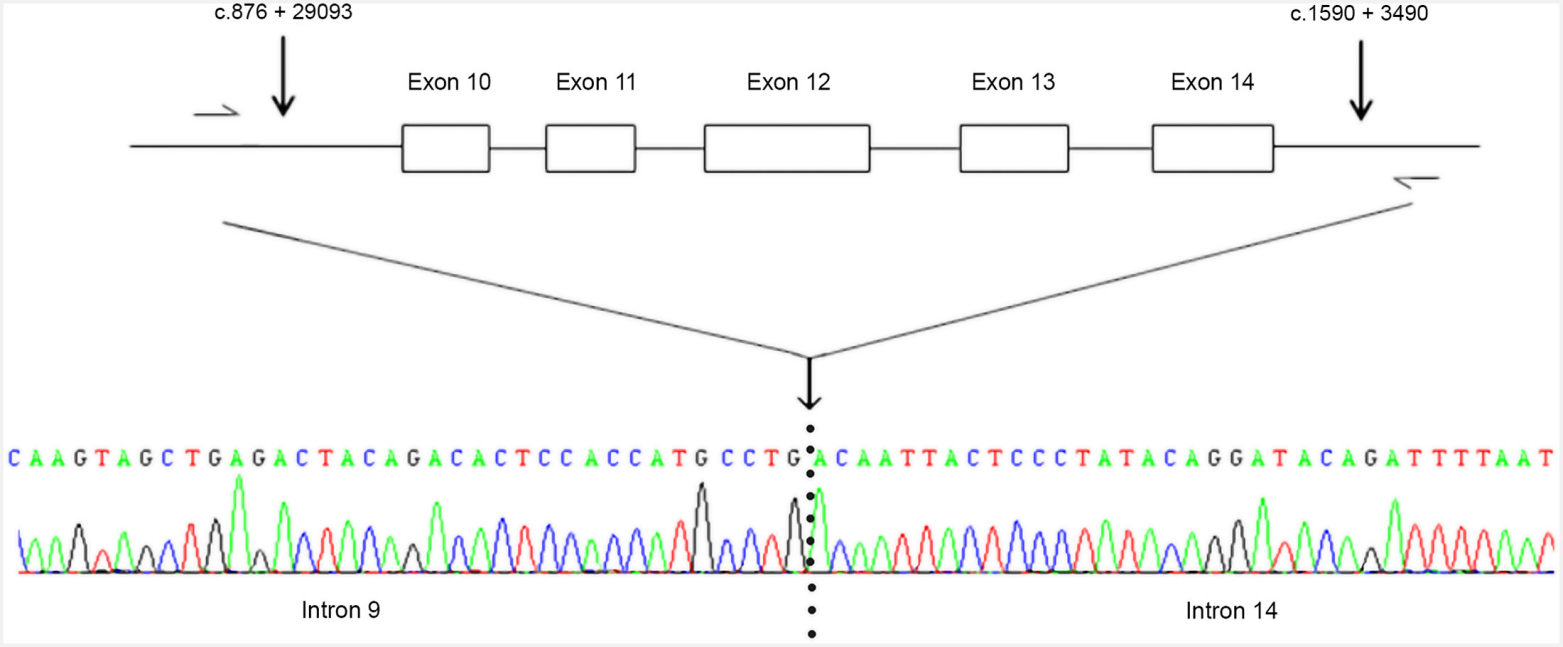
DNA sequence electropherogram showing the boundaries of the large deletion identified in *PCDH15*. The PCR intronic primers used to define the boundaries of the deletion identified in the USH1F patient were PCDH15-intron9Forward (5'-GAAATCCCTTGACCCCTTGT-3') and PCDH15-intron14Reverse (5'-GCAAAGACTTGGAACCAACC-3'), leading to an amplicon of approximately 1.2 Kb.

Finally, a biallelic deletion encompassing *PCDH15* exons 10 to 14 (p.Glu293_Gln530del) was identified in the USH1F patient, and confirmed by quantitative PCR analysis of these exons (data not shown). This large in-frame deletion is expected to affect protocadherin-15 EC3-EC5. The precise boundaries of the deletion were established by PCR amplification on the patient’s genomic DNA with specific primers in introns 9 and 14, followed by Sanger sequencing of the amplicon (Fig 4).

These results highlight the genetic heterogeneity in Algerian USH1 patients, even though *MYO7A* and *CDH23* are predominant causal genes, each being involved in 6 out of 16 patients in our series. It is noteworthy that the proportion of USH2 patients among hearing impaired children is most probably underestimated because clinical diagnosis is usually not made before the onset of the visual loss, owing to the absence of vestibular symptoms in this clinical subtype. USH2 patients were indeed underrepresented in our series of USH patients, most of whom were below 15 years old (see Table 1). An additional study, focused on USH2 Algerian patients, is therefore required to determine the spectrum of mutations in these patients.

## Supporting Information

S1 Fig DNA sequencing electrophoregrams showing the point mutations and small deletions identified in 17 of the 18 patients (see Fig 4 for the large biallelic deletion of *PCDH15* identified in the other patient). Reference electrophoregrams and sequences are shown on top of the variant electrophoregrams. Asterisks indicate the positions of the point mutations. The positions of the deletions are indicated by frames in the reference sequences, and by vertical bars on the variant electrophoregrams. Note the four electrophoregrams showing point mutations at the heterozygous state that correspond to the two compound heterozygous patients (see Table 1).
(TIF)

S2 Fig Sequence alignment of the 27 EC repeats of human cadherin-23. (DOCX)

S1 Table Primers used to amplify the exons of the USH genes. (DOCX)
