# Supplementary figures and images for: Diversity of the Genes Implicated in Algerian Patients Affected by Usher Syndrome
Source: PLoS One. 2016 Sep 1;11(9):e0161893. doi: 10.1371/journal.pone.0161893 (PMC5008642; doi:10.1371/journal.pone.0161893)

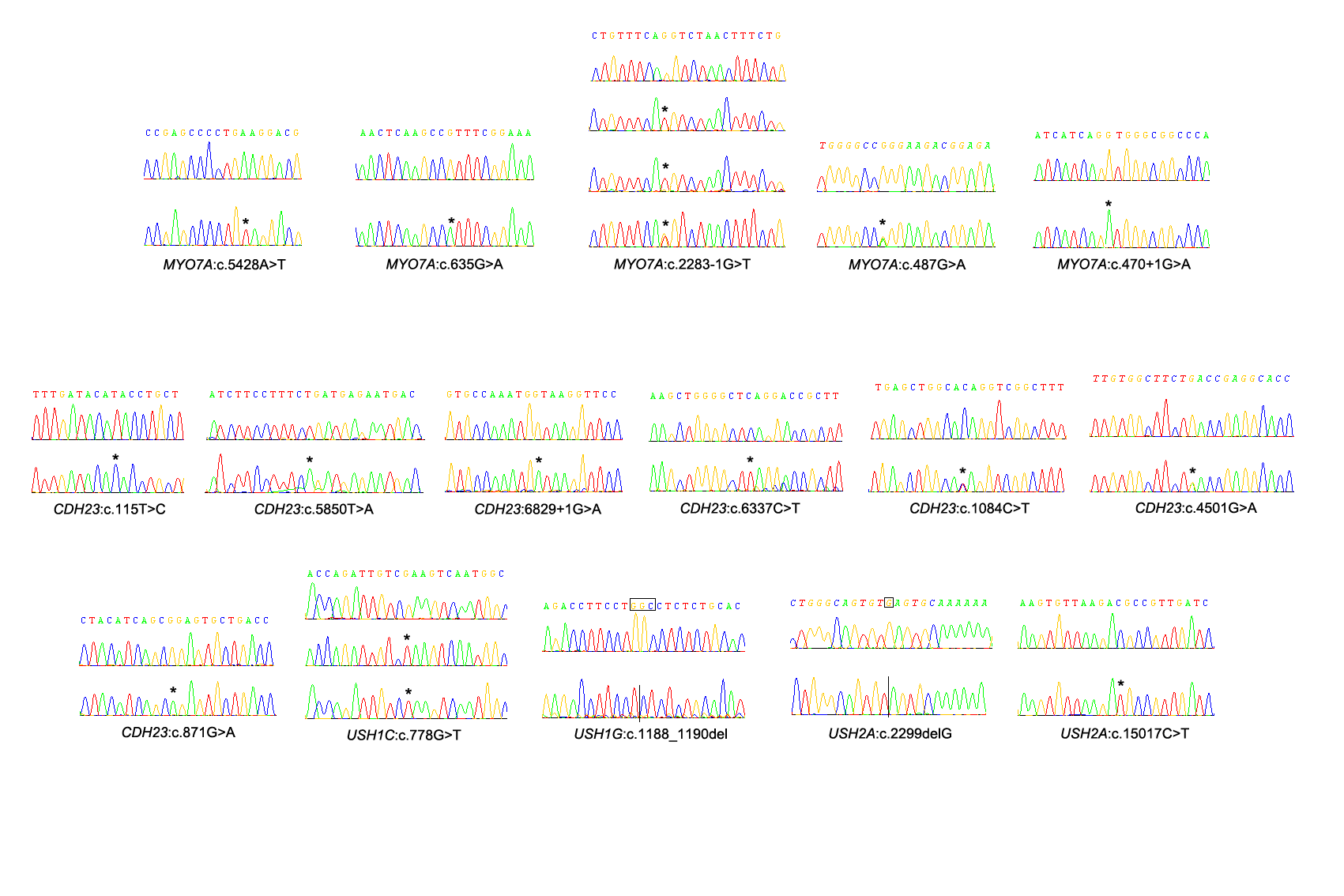

Supplement: S1 Fig — Reference electrophoregrams and sequences are shown on top of the variant electrophoregrams. Asterisks indicate the positions of the point mutations. The positions of the deletions are indicated by frames in the reference sequences, and by vertical bars on the variant electrophoregrams. Note the four electrophoregrams showing point mutations at the heterozygous state that correspond to the two compound heterozygous patients (see Table 1). (TIF) [file pone.0161893.s001.tif]
