# Supplementary material for: Diversity of the Genes Implicated in Algerian Patients Affected by Usher Syndrome
Source: PLoS One. 2016 Sep 1;11(9):e0161893. doi: 10.1371/journal.pone.0161893 (PMC5008642; doi:10.1371/journal.pone.0161893)
Supplement: S2 Fig — (DOCX) [file pone.0161893.s002.docx]

Supplementary figure S2: Sequence alignment of the 27 EC repeats of human cadherin-23

EC1  V----AWLLVLISGCWGQVNRLPFFT-NH---FFD---TY-L-LISEDTPVG---SSV--T-Q--LL-AQDMDN--D-
EC2  T----RKVNIQV---GDVNDNAPTFH-NQ---P-----YS-V-RIPENTPVG---TPI--F-I--VN-ATDPDL--GA
EC3  LSTL-ANLAIII---TDVQDMDPIFI-NL---P-----YS-T-NIYEHSPPG---TTV--R-I--IT-AIDQDK--GR
EC4  AT-VTTTFNILV---IDINDNAPEFN-SS---E-----YS-V-AITELAQVG---FAL--PLF--IQ-VVDKDENLGL
EC5  VG-Y-AKVKITL---INENDNRPIFS-QP---L-----YN-I-SLYENVTVG---TSV--L-T--VL-ATDNDA--GT
EC6  T--T-GRVRINV---LDVNDNVPTFQ-KD---A-----YV-G-ALRENEPS---VTQL--V-R--LR-ATDEDS--PP
EC7  T----VPVTIEV---FDENDNPPTFS-KP---A-----YF-V-SVVENIMAG---ATV--L-F--LN-ATDLDR--SR
EC8  GI---ATVNITL---LDINDNHPTWK-DA---P-----YY-I-NLVEMTPPD---SDV--T-T--VV-AVDPDL--GE
EC9  TS-S-ATVFVNL---LDLNDNDPTFQ-NL---P-----FV-A-EVLEGIPAG---VSI--Y-Q--VV-AIDLDE--GL
EC10 ST---STLTIHV---LDVNDETPTFF-PA---V-----YN-V-SVSEDVPRE---FRV--V-W--LN-CTDNDV--GL
EC11  TG-T-ATVFVTV---LDVNDNRPIFLQ-SS--------YE-A-SVPEDIPEG---HSI--L-Q--LK-ATDADE--GE
EC12  SS---VRVIVYV---EDINDEAPVFT-QQ---Q-----YSRL-GLRETAGIG---TSV--I-V--VQ-ATDRDS--GD
EC13  G--F-CSVYITL---LNELDEAVQFS-NA---S-----YE-A-AILENLALG---TEI--V-R--VQ-AYSIDN--LN
EC14  S--T-VKVYITV---LDENDNSPRFDFTS---D-----SA-V-SIPEDCPVG---QRV--A-T--VK-AWDPDA--GS
EC15  KK-D-HILQVTI---LDINDNPPVIES-PFG-------YN-V-SVNENVGGG---TAV--V-Q--VR-ATDRDI--GI
EC16  SA-T-THVYVTI---VDENDNAPMFQ-QP---H-----YE-V--LLDE-GPDTLNTSL--I-T--IQ-ALDLDE--GP
EC17  TS-T-TTVLVNV---NDINDNVPTFP-RD---Y-----EGPF-EVTE----GQPGPRV--W-T--FL-AHDRDS--GP
EC18  SS-T-MLVGIRV---LDINDNDPVLL-NL---P-----MN-I-TISENSPVS---SFV--A-H--VL-ASDADS--GC
EC19  RD-Y-DLLLIFL---SDENDNHPLFT-KS---T-----YQ-A-EVMENSPAG---TPLTVL-NGPIL-ALDADQ--DI
EC20  S--T-AHLLITI---LDDNDNRPTFS-PA---T-----LT-V-HLLENCPPG---FSV--L-Q--VT-ATDEDS--GL
EC21  GT---AIVTILI---DDINDSRPEFL-NP---I-----QT-V-SVLESAEPG---TVI--A-N--IT-AIDHDL--NP
EC22  -P-N-AKLTVNV---LDVNDNTPQFK-PFGI-T-----YY-MERILEGATPG---TTL--I-A--VA-AVDPDK--GL
EC23  AE---IPVYLEI---VDINDNNPIFD-QP---S-----YQ-E-AVFEDVPVG---TII--L-T--VT-ATDADS--GN
EC24  -S---VQVVIQV---LDVNDCRPQFS-KP---Q-----FS-T-SVYENEPAG---TSV--I-T--MM-ATDQDE--GP
EC25  G--T-TMLLVEV---IDVNDNRPVFVR-PPNGT-----I--L-HIREEIPL---RSNV--Y-E--VY-ATDKDE--GL
EC26  M----QPLQVAL---EDIDDNEPLFV-RP---PKGSPQYQLL-TVPEHSPRG---TLV--G-N--VTGAVDADE--GP
EC27  --------RVVL---EDINDQPPRFT-KA---E-----YT-A-GVATDAKVG---SEL--I-Q--VL-ALDADI--GN


EC1   ---P--LVFGVS---G----------------------EEAS-RFFA-VEPDT----G----V-VWL-R--QPLDRET
EC2   G-GS--VLYSFQ---P------------------------PS-QFFA-IDSAR----G----I-VTV-I--RELDYET
EC3   P-RG--IGYTIV---S-----------G-----------NTN-SIFA-LDYIS----G----V-LTL-N--GLLDREN
EC4   N-SM--FEVYLV---G-----------N------------NS-HHFI-ISPTS--VQGKADIR-IRV-A--IPLDYET
EC5   F-GE--VSYFFS---D-----------------------D-P-DRFS-LDKDT----G----LIMLI-A--R-LDYEL
EC6   N-NQ--ITYSIV---S-----------A-----------SAFGSYFD-ISLYE--GYG----V-ISV-S--RPLDYEQ
EC7   EYGQESIIYSL----------------------------EGS-TQFR-INARS----G----E-ITT-T--SLLDRET
EC8   N-GT--LVYSIQ---P-----------P-------------N-KFYS-LNSTT----G----K-IRTTH--AMLDREN
EC9   N-GL--VSYRMP---V-----------G-----------MPR-MDFL-INSSS----G----V-VVT-T--TELDRER
EC10  N-AE--LSYFIT---G-----------G-----------NVD-GKFS-VGYRD----A----V-VRT-V--VGLDRET
EC11  F-GR--VWYRIL---H-----------G-----------NHG-NNFR-IHVSN----G----L-LMRGP--RPLDRER
EC12  G-GL--VNYRIL---S-----------------------GAE-GKFE-IDEST----G----L-IIT-V--NYLDYET
EC13  ---Q--ITYRFNAYTS-----------T-----------QAK-ALFK-IDAIT----G----V-ITV-Q--GLVDREK
EC14  N-GQ--VVFSLA---S-----------G-----------NIA-GAFE-I-VTTNDSIG----E-VFV-A--RPLDREE
EC15  N-SV--LSYYIT---E-----------G-----------NKD-MAFR-MDRIS----G----E-IATRP--APPDRER
EC16  N-GT--VTYAIV---A-----------G-----------NIV-NTFR-IDRHM----G----V-ITA-A--KELDYEI
EC17  N-GQ--VEYSIM---D-----------G-----------DPL-GEFV-ISPVE----G----V-LRV-RKDVELDRET
EC18  N-AR--LTFNIT---A-----------G-----------NRE-RAFF-INATT----G----I-VTV-N--RPLDRER
EC19  Y-AV--VTYQLL---G-----------------------AQS-GLFD-INSST----G----VVTVR-S-GVIIDREA
EC20  N-GE--LVYRIE---A-----------G-----------A-Q-DRFL-IHLVT----G----V-IRVGN--ATIDREE
EC21  ---K--LEYHIV---GIVAKDDTDRLVP-----------NQE-DAFA-VNINT----G----S-VMV-K--SPMNREL
EC22  N-GL--VTYTLL---D-----------L-----------VPP-GYVQLEDSSA----G----K-VIA-N--RTVDYEE
EC23  F-AL--IEYSLG---D-----------G-------------E-SKFA-INPTT----G----D-IYV-L--SSLDREK
EC24  N-GE--LTYSLE---G-----------------------PGV-EAFH-VDMDS----G----L-VTT-Q--RPL--QS
EC25  N-GA--VRYSFL---K-----------T--------AGNRDW-EFFI-IDPIS----G----L-IQT-A--QRLDRES
EC26  N-AI--VYYFIA---A-----------G-----------NEE-KNFH-LQP-D----G----C-LLV-L--RDLDRER
EC27  N-SL--VFYSIL---A-----------IHYFRALANDSEDVG-QVFT-MGSMD----G----I-LRTFD--L-FMAYS


EC1   -K--SE---FTVE--FSV--SDHQGV------------------------------------------I--------
EC2   -T--QA---YQLT--VNA--TDQDKT--------------------------------R---------P--------
EC3   -P--LYSHGFILT--VKG--TEL---------------------------NDDRT--PSD-----------------
EC4   -V--DR---YDFD--LFA--NESVP--------------------------D----------------H--------
EC5   -I--QR---FTLT--IIA--RDGG------------------------------GE-----------E---------
EC6   -I--SN---GLIYLTVMA--MDAG------------------------------NP-PLN-------S---------
EC7   -K--SE---YILI--VRA--VDGGVG--------------------------------HN------QKT--------
EC8   -P--DP---HEAE--LM-------------------------RKIVV-SVTDCGRP-PLK--------A--------
EC9   -I--AE---YQLR--VVA--SDAGT-------------------------PT------K---------S--------
EC10  -T--AA---YMLI--LEA--IDNGP------------------------VGK------R---------H--------
EC11  N---SS---HVLI--VEAYNHDLG ------------------------------- ---PM--R-------------
EC12  -K--TS---YMMN--VSA--TD------------------------------QAP--PFN----------------Q
EC13  -G--DF---YTLT--VVA--DDGG------------------------------P--KVD-----------------
EC14  -L--DH---YILQ--VVA--SDRG------------------------------T--PP--------R---------
EC15  Q---SF---YHLV--ATV--EDEG------------------------------TP--T---L--------------
EC16  SH--GR---YTLI--VTA--TD------------------------------QC---PIL--S--HRL---------
EC17  -I--AF---YNLT--ICA--RDRG------------------------------M--PPL-----------------
EC18  -I--PE---YKLT--ISV--KDNPE------------------------NPR------IA--------R--------
EC19  -FSPPI---LELL--LLA--EDIG------------------------------LL-----------N---------
EC20  -Q--ES---YRLT--VVA--TDRG------------------------------TV-PL--------S---------
EC21  -V--AT---YEVT--LSV--ID------------------------------NASDLPERSV-S------------V
EC22  -V--HW---LNFT--VRA--SDNG------------------------------SP-PR--------A---------
EC23  -K--DH---YILT--ALA--KDNPGD-----------------------VAS---N-RRE--------N--------
EC24  -Y--EK---FSLT--VVA--TDGG------------------------------EP-PL--------W---------
EC25  Q---AV---YSLI--LVA--SDLG------------------------------QPVPYE--T--------------
EC26  -E--AI---FSFI--VKA--SS------------------------------NRSWTPPRGPSPTLDLVADLTLQEV
EC27  -P--GY---FVVD--IVA--RDLAGHNDTAIIGIYILRDDQRVKIVINEIPDR-VR-GFE------EEF--------

The Tyr39 and Asp1501 residues are highlighted in red.

Sequence alignments were performed using T-coffee (http://tcoffee.crg.cat/apps/tcoffee/do:expresso)
