# Supplementary material for: Diversity of the Genes Implicated in Algerian Patients Affected by Usher Syndrome
Source: PLoS One. 2016 Sep 1;11(9):e0161893. doi: 10.1371/journal.pone.0161893 (PMC5008642; doi:10.1371/journal.pone.0161893)
Supplement: S1 Table — (DOCX) [file pone.0161893.s003.docx]

| **Gene** | **Exon #** | **Forward primer (5'-3')** | **Reverse primer (5'-3')** |
| --- | --- | --- | --- |
| *MYO7A* | Exon 1 | AGCAGTGCTGGCTGGACA | ACATCCAAACCCCTCCCAAT |
|  | Exon 2 | CTTTGGGAGGAGCCCAG | GTTTGGAAGCCTAGGCAGG |
|  | Exon 3 | GCACTGCTCCAGAAGGTTG | ATCTGAGAGGCAGAAGTGGG |
|  | Exon 4 | AGAGAGGTCGAGGCCCTTAC | CACAGCGGACAAAGTCTCAG |
|  | Exon 5 | TGACTCCAAAGCCAGGACTC | TTCCTGAAGAGCTTGATGGC |
|  | Exons 6&7 | TGGAGGGTCCGTATTGTCAG | TCCAGCACAGAGGGAAGC |
|  | Exon 8 | GATGGGCTTCTCCACAAGCCATTTG | AGTCTGCAGTAGCCAGCTCCCTAC |
|  | Exon 9 | AGGAGTTGTGGTGCTCACCGGGTG | ACCGCTGATTTCAATCTAGCAGAC |
|  | Exon 10 | TGATGCCCTCCCTGGAC | TGGTTGCTTCTCCATATTGAAC |
|  | Exon 11 | CGTGCTTAGTGGAGGCAG | ACTCAAGGAAGGCCGACTG |
|  | Exon 12 | GGTTTCACACGGCACTTTG | AGGAAAGGGAGAGGGTCCAC |
|  | Exon 13 | CTCGCCTCTGAGTTTGGAGTCCATG | AGCAGGGAAGGAAGCTGTG |
|  | Exon 14 | GTAGTTCCAATTCATCCACTTAAC | ATGCACAGCACTGTGAAGTACTTAG |
|  | Exon 15 | CATGAAATGGATGTGGTGGA | ACAAGGGCAGGGCTTAGACT |
|  | Exon 16 | GAAGGTGGGGAGAGGCAGTTTG | TGCTGCCTCCAATAAATAGGGGG |
|  | Exon 17 | CCTGTCCCAGCTTTGCTC | TGCACCTGAGAGGTCAAGC |
|  | Exon 18 | CCCAGCTGAGGTCACACTTC | CTCCTCCAGCCACACCAC |
|  | Exon 19 | GGGCCCAACTGAGTTCTTG | TGTGCACTTGTTTCATAGGTG |
|  | Exon 20 | AGAAGTTATGTGCCTTGCCC | CAGGGCAGTGGCAGGAG |
|  | Exon 21 | GGGGTGCCTGTCTGAGAGTGG | GAAATGAGGGTCTGGTCCCAGG |
|  | Exon 22 | TGACCTAGAGAATTTCTTGATTACC | AGAGCTCAGGCAATGCTGG |
|  | Exon 23 | AGAGGCTCCATTCTTCCCAG | ATCTCTGCTCCCCACTGTTC |
|  | Exon 24 | GGTCGTACCCTGTTGCTCAG | CAGACCTGGTCCCTGGC |
|  | Exon 25 | CCAGGGACCAGGTCTGACTA | GTTCCCACAAGTAGACAAAGG |
|  | Exon 26 | GAGCAGGTGGACGGTGG | ACCCCTGAGGACAGACAGTG |
|  | Exon 27 | TGACAGTGATGGGGAGCC | AACTAGCTAGCAGCGAAGCC |
|  | Exon 28 | GGTGGTCTTGGCAAGTTGG | GAAGATGGGGCTCAGTAAGG |
|  | Exon 29 | TAGGGAAGCCACAGAAAGCAACCTG | TCCAGAACTTGAGGGTAGGTGTCTG |
|  | Exon 30 | GCAGGCAGGTCTGAAGGG | AAGTAGGTGGCAGCACTGG |
|  | Exon 31 | CTTGGACGTGTCCCTGC | AAGAGTGCCTGGTTGCAGTC |
|  | Exon 32 | AGAAGCCTGGGTCCCAAAG | AGGAAGGGCCACTGGAAG |
|  | Exon 33 | ATGCACAGGAGGTGCAGG | AATTTGTAGGCTGTGGGCTG |
|  | Exon 34 | CACGTAGCGAGTTTGTGCTC | CCAATCACGTGCAACCAG |
|  | Exon 35 | GGCATGACTGACTCAACTGG | CATAAGCCACCGTGCCC |
|  | Exon 36 | TGACAAGGTGGAGGCAGAGTGG | GTTCCTCATCTGATGCCATTTCTAGG |
|  | Exon 37 | GGCCACAGGTAGAGAGCTGA | GACCAGACAGTAGCGGAAGC |
|  | Exon 38 | ACACAGGGATGGGTGGG | CAGGCCCTCAGGAGGAAG |
|  | Exon 39 | CCCATTTTGTGAGTGTGCAG | CTCCCGGCTCTGTGTGAG |
|  | Exon 40 | AGGTCCTGTGACTCCCGATG | AGGGGCTCATCCCACAAG |
|  | Exon 41 | GTCCCCTGGTCTCCACAGT | AGAGGCTTAAGGCCACCTTT |
|  | Exon 42 | TTAAATGCCATGCCCAGCTC | AGGGATGGAAGAAGATGGCC |
|  | Exons 43&44 | GACACACAGAAACCCCTTCC | AATCCCTTTGAGGCCAGG |
|  | Exon 45 | GCCTGTGACCTGCTCTGTCT | ATTCAGGGCCCCACCATT |
|  | Exon 46 | TCTGCTGTCCTAGTCCTGGC | AGGGCAGGTGTCCAAGG |
|  | Exon 47 | GGGCCGTCAGTACCACATAG | ACGTGATGGGTGGATGAGTC |
|  | Exon 48 | GGCACCTGCTTCCCACCGCAGCTG | TGTGTTTGTTCCAGGCCCTGGGAGGC |
|  | Exon 49 | AGGAGGGATTTGCCTTTGTC | TATAGTCAGTGGTGGCTGGC |
| *USH1C* | Exon 1 | GGCTCTTTCCAGCTCCTG | GAAAAGGGAGGGGCAGTG |
|  | Exon 2 | CCTTGGGGATGACCTCTC | CCTGAAGACAAAGGAACCC |
|  | Exon 3 | CTGAGGTCATGGCCAGGG | CCAGGGTGATCTCTCCACC |
|  | Exon 4 | GTGCAGAGGAAGCCACCAGGCTGG | TGGGTGACCATGTTGTGCCACAC |
|  | Exons 5&6 | CATCTGGTGGTGAGTCTGCC | ACTGGCCCAGAAGCAGG |
|  | Exons 7&8 | GATGTGGGGCCTGGTTC | GAAGGGGAGGGCAATAGG |
|  | Exon 9 | ATCCTGAGCACTGGACATGG | GATCACAGTCTGACCCAATTTC |
|  | Exons 10&11 | TCCTGCAGATGAGCGAGTG | TGTGTGGCAGAGATCAAAGG |
|  | Exon 12 | CAGGGGTGTGTCAGGGG | AGGAAGCAAGCTAGGTGGG |
|  | Exon 13 | TCCAACTTTTCTCCAAATCAAG | AACTGGTTCTGGAGGCAGC |
|  | Exon 14 | CTCCGATATGCAGGAGATGG | AGGGAGGGCCAGCATTTC |
|  | Exon 15 | GGATGCTCTGGCTGAGCTG | AGGAATGTTTGGGAAAAGCC |
|  | Exon 16 | GCTGTGCTGTCGACACTATTTC | AGCTGGTCCTCTGGGAGC |
|  | Exon 17 | TGCTTTCATGCTATTTCTATCCAC | CCACAGTGGGTCATTCTGAAG |
|  | Exon 18 | GGTGGGTCTCCAGTAGCAAG | GAAACAGCAGTTCTCCCCAC |
|  | Exon 19 | TGTGTAGAAGTAGCATGGCTGG | AGGTAGCCCTGGTCTGCTC |
|  | Exon 20 | GCAGCCAAGACCTATTGGTAAC | CCCAGAGAGAAAGAAGTGGC |
|  | Exons 21&22 | CTCTGACCCTCAAGACCCTC | GTAGAGGCAGGAGACCAAGG |
|  | Exon 23 | TCAAGAGACTCCTTGGTGCC | GCCCCGTCTAACCACTGTATC |
|  | Exon 24 | CCCTTTGCCAAGCCCTG | GGGTTTGAGGCAGGCAG |
|  | Exon 25 | GAAATGGGGATGGAGTGC | AGGTGGCTGAGATTCTGGAG |
|  | Exon 26 | TCTGCCTGGGAGTAACCAAG | CAGATGGCCACTGCAAGC |
|  | Exon 27 | CTCTGGCTGGGCTGAGTG | AGTGTGGCCTCTCTCAAGGC |
| *CDH23* | Exon 1 | AAGTTGATCGCGGACTTGAG | GAGGGGAAGGAGAGGGAAG |
|  | Exon 2 | CCTCTGCCACCCAGGAAG | GAACCACCAGCCCCAAC |
|  | Exon 3 | AGTGTGTAAAGCTGATGGGG | TCACTTGTGGGAAGGGATTG |
|  | Exon 4 | AGGAAGGCATAAACGTGACC | CCAGTGTCTCCTCCTGTCCC |
|  | Exons 5&6 | AGGACCCAGGACCTCAGC | ACCATGGTGGTCTGGGG |
|  | Exon 7 | GCTTTGAGGGACTCAGCC | CACTCTCTTCCAGGGTGGG |
|  | Exon 8 | ACGGTGCAGGTCTGTGTG | GAGCCCAGCCCACTAACAG |
|  | Exon 9 | AGAAAGACAGCAGCCAGGG | GAGCCTGAGATGCCTACTGG |
|  | Exon 10 | TGATGAGTCTTTAATGCCCAGAGAGG | ACGGAAGCGAAGTGCTTCCGGCGCC |
|  | Exon 11 | AAGAGCTGTTGCTGTGATTAGC | GAGGACCTGAACAGCCCAG |
|  | Exon 12 | CATTTGGGTCTAGGCTTTGG | TTCTACCCTACCCTCGCCTC |
|  | Exon 13 | AGGGACAAGGACTCTGGGAG | ACAAAATCCCTCTGGGTGC |
|  | Exon 14 | AGGAACATGGGTTTCCTGTG | AGGGCCAAGGACCACTG |
|  | Exon 15 | GGCCCCATCAACAAGCC | GCAAGCAAGCAAGTACAGGG |
|  | Exon 16 | AGCTGTGAACAGGGCCAG | GTGTGAGTGCAGAGAGCCC |
|  | Exon 17 | GACGGGCATCTCAAGTCTG | TGGGTTCACAGGAAGCACTC |
|  | Exon 18 | TGCCCACCTTAGACATCTGG | GTGACTGCTGGCTGCCTAC |
|  | Exon 19 | CAAATGCTGCTCCCAGG | GGACAGTCAGCCTCAGGG |
|  | Exon 20 | CCCTCCCTCTCTCCCTGG | GGGGTCCAGAGGTACAAGC |
|  | Exon 21 | ATCTGAGCACTTTTCCCCTG | CACAGACCCCTGGGAAGG |
|  | Exon 22 | AGAGACACCCTCCCCAGG | CAGAGGCCCAGTCACCAG |
|  | Exon 23 | CCACTTGCCTTCTTCCTGTC | GCTCCTCAGGCACCCAC |
|  | Exon 24 | GCATCCATCCCAGTGTCC | GACATGGGCTCCCCTCTAC |
|  | Exon 25 | CTCTGGAGCTGGGTCTTCTG | TGGCTGTCCATCTGGGAG |
|  | Exon 26 | AGCAGAGTCCCCGCTTC | ACCCTGTGTGAACTCTGCCC |
|  | Exon 27 | GGAAGTGTGCCCCTCTCTC | CACTGAGCATGTGCCCTC |
|  | Exon 28 | GTAGGATGCGTGAAGGGAAG | TTGGCAGCCTAGAGAAGCTC |
|  | Exon 29 | CCAGCACAGCAGGAGACTTC | CTGTTCTAGCTGTGGGCTTG |
|  | Exon 30 | ACACAAGGCGGCCACAG | TGGGGAGGTTTGCTCTG |
|  | Exon 31 | GGCAGCTTGAGAAGCCAC | CAGAAGGAGCTCAACCAATG |
|  | Exon 32 | AAAGTGGGCAGAATGACCAG | GGAAGTGTCATGGCCAGC |
|  | Exon 33 | CCATTTGCATCTTTGCCTTT | GTGTGAGAGTGGAGGCACTG |
|  | Exon 34 | ACGGTGGGACCCAGGTG | CTGTGAGAGCTGTGGGAGC |
|  | Exon 35 | ACCTGGCCACCTCTCCTC | GAGTAGTGACCGGTCTAGGCAC |
|  | Exon 36 | CTCCATATTCCCAATCCTGC | GCAGACATTTAAACAGGGGC |
|  | Exon 37 | CCAGACTGTGCCCCAATG | CTCACCCATCTTGTTCCCC |
|  | Exon 38 | TGTTTGAGTCACATGGAGTGAG | AGACTGCATCTTTGCTCCATC |
|  | Exon 39 | AAATTGGTCAGAGGGTGCTG | CTTTCTGGGTCCCATCCTTC |
|  | Exon 40 | CAGCACAAAGCTGGCTGG | TCCCTTCTTATCCTCCCCTG |
|  | Exon 41 | GAAGCAGAAGGAGTCCTGGG | CTCTGTGGCAAGTACAGGGG |
|  | Exon 42 | CTCCTCGGTTGCCATGC | AGCCTCTAGGGCAGGGTCC |
|  | Exon 43 | AGCAGTTGGCATCTGTGGG | ATGTGTTCTCTCTCCATGCG |
|  | Exon 44 | CACCTCTGTGCCCCAATC | GTTCAGCTATGCCGTTAGGC |
|  | Exon 45 | ATGGCTCACCGGGACAG | CTGGGTCCAGCACTGAGAAG |
|  | Exon 46 | TGGGGGAGAAAGTCATTTTG | AACTCCGTGTCCAACCTGAG |
|  | Exon 47 | GGCTGTCATGAGAAGGGG | GGAGACAGGACTGGGAGC |
|  | Exon 48 | CTGGGAAGGGCAGATTTTAG | CCTGCCCTGATTGGCTC |
|  | Exon 49 | TGGCTGAGGCTAAGGAAGG | TGGGGACTAAGCAAGCAGAG |
|  | Exon 50 | GAGAGCTGCCATGAATGAGG | GGAGAGGAAGATGCCCAAAG |
|  | Exon 51 | CGGGCTCTGGGACTGAC | ACAATACTTTTGGTGGCTGC |
|  | Exon 52 | CAGCATCTGGCCATAGTAGG | TGCTTGTTGCCTCCACAAC |
|  | Exon 53 | GAGGCTGCCCCATCCTG | CACGCTTCCCTCTACTCCTG |
|  | Exons 54&55 | GGAAGCGTGGGAAGGATG | TTAGAGTGGGGTGCTTCCAC |
|  | Exons 56&57 | CAGGAGAAAGAGCAAGGGC | GGCAGGTGAGTCCCTGTG |
|  | Exon 58 | CTGAAACAGGGACTGGAAGC | GAGGCAGGTGGTGTAAGTGG |
|  | Exon 59 | CAGGTGCAGGGACTGGAG | CCATGCAGTGACTGAGAGGTC |
|  | Exon 60 | GGATTCGGGGCACTGAG | CTGTCTCTCCTTGTGGCCTG |
|  | Exons 61&63 | CTAAAAGAGGCCTGCCCATC | CTACTCCAGGGCAGGGAGG |
|  | Exon 64 | GAGCAGACTGTCGGTGGTG | GGTGGGCACTCCAGGTC |
|  | Exon 65&66 | CCCTACCCCTGGCTATGC | AGACTGCAGCTCTCCCCAG |
|  | Exon 67 | GAAGGGCACCTGTCTACTCG | CTGTGTTCTCTGGAGTGGGG |
|  | Exon 68 | CCCCTTACTCCCAGAGGG | AGAGAGCATCCCCAGGC |
|  | Exon 69 | CCACATAGCCAGTGGGTCTC | GGAGGGGACGGTGGATG |
| *PCDH15* | Exon 1 | CTCCTGTATGTGCTGCGAGA | TGTGGTTCACTGCTGCCTTA |
|  | Exon 2 | AAGGCTTTTCTGTGCTGTGAC | GGACATCTGATTTCACTTTTCTAAGC |
|  | Exon 3 | ATGCACAAAGTGACTGTATTATGATGTG | ACCCATACTGGAGGGGAATTATCC |
|  | Exon 4 | AAACTGATTGTGAGCCAGCC | AAATGAGTTAAAGAAACTGTTGTTGC |
|  | Exon 5 | TGAACGAGTGCTTTGACAGTG | TCGGAAGTCACATACTTCTTCTG |
|  | Exon 6 | AACATTTCTGGTGTGCAGTTG | TGGTTCTGGAAGTTACTGAATAATAC |
|  | Exon 7 | TGTCATCTCTAACTTCCAAGAGG | TGCCCTGATGAAGAAGTGAG |
|  | Exon 8 | ACTGCTCCTGGCTATTGTGC | AAAATAAAATAACCATGTTGGACTG |
|  | Exon 9 | TTTGAGAATACTTTGCTCCCC | TTCCTTGGAATTGAGAGAATTTG |
|  | Exon 10 | GTCTGCTTTCCCTGAACTGC | AAGTGGGCTTCGCTTATTTTC |
|  | Exon 11 | TTGCATGAAAAGTGAAACTCC | GCCATATCTTTGTCATATTTCCC |
|  | Exon 12 | TTCCACGATATAGGTCACCATC | AACCAGACATCTCTTTCAGTTCC |
|  | Exon 13 | AAAATATGCCTGGAGTTGAATACC | TGGCGGATATGCTTTTATTTG |
|  | Exon 14 | CGACATGGTGGTGAAAAGTTG | AATTACAGGGTTAAACGGGC |
|  | Exon 15 | AACCCAACAACTGAATAAACAATG | TGAGGTTTCTCCCTCCATACAC |
|  | Exon 16 | TTACTCTGTAATGAGTGGGCG | CAAACAGCTGAAAGCCTCTG |
|  | Exon 17 | AAGCTGTGATTACTGAGAGGGG | CATTAATTCATGTCTGTGATACATTG |
|  | Exon 18 | AAATGCCAGAATGACTACTTAAATG | AAAGTCTTGAAGAATATCCAGCAC |
|  | Exon 19 | CCTTCATTTACAGTAGCCCGTC | TAATTTTGGCACACAAACCC |
|  | Exon 20 | TTCCATCCTTCCTGCATTTC | TGGGTCTGGTACCACTGTGTC |
|  | Exon 21 | AACTGTATCCCTAACCTGGTTTC | CAGAGACTACTTTTGCTGTCTTGTG |
|  | Exon 22 | CATTGCACTGTGTTTTAACTTGG | TGTGCTGTCATCTGTTAAGCC |
|  | Exon 23 | TTCTGGAACCAGGCAATCC | CATCAATTTTGAGAGACTCCTTTC |
|  | Exon 24 | ATGAATGTGTGAGGGCAGAG | TTAGGCCAAATCTAAGTTTACTGG |
|  | Exon 25 | AGCCAGCATTTTGTCACTTTC | AACGAATAGGCAATCATCTCAG |
|  | Exon 26 | TGGCTTTTGATTTATTTTCAATG | CTCCAAGTTTCAGGCTTTTG |
|  | Exon 27 | TTCATAAGGGTTATCTTTTCCTGC | CTGAAAACACTGACCTATGGC |
|  | Exon 28 | GCATAGTAGGGAAGAGGTAATGTG | TCGAAGCCTAAACCTCACC |
|  | Exon 29 | TCATTGGGAGCTAAATGCTG | GCCCTCTGAAGTCACTGTAGC |
|  | Exon 30 | GACTTCTGACTCAGCTTGTGTTTC | ACGCAAAGCCATGTTACCAG |
|  | Exon 31 | TGGCTTGAATGACTCTAACGG | AGATGTGGTTCTGAGCAGGC |
|  | Exon 32 | AATGATACCCTGTGGGGATG | TTCAACTTAACCTTTCTTAGATTTTCG |
|  | Exon 33 | TCTTTCTCATGAAAGCTGTGTC | GCCACTGCTGCAGATCTATG |
|  |  | GGGCCCAAGAGAAAAGATTC | TCTTTCGGTTTCAATAGGTAACATAC |
|  |  | TTCAAGAACTGTGGAACTCAAATC | TTGGTCGTGCATTTAACACC |
|  |  | CCAACTCCTCTTCCTCCAC | GCATTTCATTGAATTTGGGG |
| *USH1G* | Exon 1 | GGGTGAGCGTTTCAGATGTCTTG | GGCAGCTCAGAGGAGTGGTGGA |
|  | Exon 2 | TCCACCTCCATCCCCAG | CGTGGCCTGAGAGTACGG |
|  |  | GACACCCTCAGCTTCTCCAG | TCCCACCCCATCCAGAC |
|  |  | ACGACTCCCTGTTTACCCG | AGGGGCCTTCCAACTCC |
|  | Exon 3 | GGGTGAAAATGATGCCTCTG | AGGACCTCGAGACCCCAC |
| *CIB2* | Exon 1 | CTCCTCCAGGCGAGCTG | AGGGTTTGAACCTGGGAGAG |
|  | Exon 2 | CTATCCCCTGTGAGCCCTG | ATGCTCTGCCTCAGCCC |
|  | Exon 3 | TGTTCTGCAGAGGCAGAGG | AGACACAAAGGGTGGAGCTG |
|  | Exon 4 | ACTCCTCCAGGGTCACACAG | CCTAGGGCAGGAGCCAGTAG |
|  | Exon 5 | AGCTGGTGGAGGTAGTGGG | ACCACTGCTCACAAATAGCG |
|  | Exon 6 | CATCCACTATGCAGGGTTCC | TGTGGTGTAAACCCCAGAGG |
| *USH2A* | Exon 1 | TCTGAGCTTCAGGTAACCAAG | CCCTAATACTGTGCAAGTAATTGTATT |
|  | Exon 2 | TTCCAGAAAATGGAGTATCGC | TGGGGAAACAACTGGAAGAG |
|  | Exon 3 | CACCACTGTAACTGCACAATACC | GAAAGGAAGACAAATCCTTGTG |
|  | Exon 4 | GTCTTCCCAGCTGAACAAAG | CCCTAGAAGATGAATACACGTAG |
|  | Exon 5 | TTGTCAGGTATTGCTTGGTAAAC | ATTTAAGTGAATTCAGCATTTATCC |
|  | Exon 6 | TCACTCTCAGTAGTTAGTTTAGCATCC | CAAGTAACACCAAGGGCCAC |
|  | Exon 7 | TTTGAATCTAATAATTCCATGGTTTG | TGGTGGTGAAGGGAAGTCTC |
|  | Exon 8 | CATTTTGATTTCTGTTTTGCC | ATGTGCTGTTAAGACAGTAAGTATGAC |
|  | Exon 9 | TTCCCTGTTAAATTCACATAATGC | TTTCAACTTAATTGATGGCTGTT |
|  | Exon 10 | TGTGCTTTACTTCTGGTGAAAG | GGAAGATTTGGTGAACTAGCTTTG |
|  | Exon 11 | ACAGTCTTTAATTTTGAATTATGCAG | AACATCCAAATAAGTTTCTGGC |
|  | Exon 12 | CTCCCTCCCTGTCTTGTACC | TTTTCCAGCCTGTCTTGAGC |
|  | Exon 13 | CCTATGGCATTGCTTGTGAG | CAGTTGCAAGGCAGACAGAG |
|  |  | CAGTGCATCTGCAAGCCC | CCAGAAACAGGGAGAAGTTACC |
|  | Exon 14 | ATTGGGAATTAGTGCCTTGG | GTTATTGCTTTGCAACTGCC |
|  | Exon 15 | AACAAGCCGTCTTACTCTACAATG | CAGTCCCCTGTATGATGCTG |
|  | Exon 16 | AGCAATCCTGAATCAGAAAGAC | CCAGACAGAGGAAACCACAAC |
|  | Exon 17 | GAAAGCAGTTAGCAATGATTCTTC | TGCCAAATTCCTAATTGCAC |
|  | Exon 18 | CAATGAATATCCAGCACTCTTTAAC | AACATTGATCTTTACCTGATTCTCC |
|  | Exon 19 | TATAGGGACGACATTCATTGTTTTG | AGAATTCTGTCACAGAATTCTGC |
|  | Exon 20 | ATTCATGGAACTTTGACTGCCAAG | GCAATCAGAGTTAGTGAGGGAGGAG |
|  | Exon 21 | CACAACTGATTTGATTTGAAGTGTG | CAGGCAAAAGCTATCAAAGGGC |
|  | Exon 22 | TGCTGTGTATAATTTCCTGACTG | TTTCTTGATTGGCAAAATGG |
|  | Exon 23 | TGTATGCATGCTTGTATCAGG | TGGAATTGAGTAGAAATTATGGTG |
|  | Exon 24 | ACAGGCAATGAGGAGAGAGG | CATAGGTCCTAAAATAACATACCTTTG |
|  | Exon 25 | GCTGATGGTGTGGTGTTTACAT | CAAGAACTGCATGAGTTACCAGA |
|  | Exon 26 | CAAAAGGAATTTCTTAACATTGATTTG | TCAAAAGATAATGAGGGAAATAGTG |
|  | Exon 27 | TCTGCTCTCTCCCAAACTGC | AAAACTGTTAGCACCAGGGC |
|  | Exons 28&29 | CAAAATGTAGTGCTGCAGAGG | GCTTCAGGGTAATAGTCCTTCC |
|  | Exon 30 | TGAGTCTGATTTACTGCGGC | GGCTTCCACTACTTTAGATGGG |
|  | Exon 31 | ACAATCAGCAGAAAGGGAGG | AATGGAGCAATTATGGCCTG |
|  | Exon 32 | CCATGATTTATTGGTTTGGGTC | GCAGATATGGAACCCCTGG |
|  | Exon 33 | GGAGGAGTGAAATCTCCTCAATAAG | TATGTCACAAGCTCCTCCCC |
|  | Exon 34 | CAAATGTGTCCTTTGAAAATCC | AGGGGAGAAGAAGTGGAAGG |
|  | Exon 35 | TTTGGGGAAGTAAAGGTAGCAC | AAAACCACAATCTCCCCAAC |
|  | Exon 36 | TCCTTTGGTAAGAGACAGAGTTTAG | AAAATCTCCCATCCTGCTTG |
|  | Exon 37 | TGAGGTGTGCTTTGATCCTG | GAGAACCGTCGGGCAGTAG |
|  | Exon 38 | GAGTGGTAAAGTTACAGACCTATTTGG | CAAGGTATTCAATGGAAACTTAATTC |
|  | Exon 39 | TCCATCCAGCATATGTTCTCC | TTGCAAATGAGAACTGACCTACTC |
|  | Exon 40 | CAAGATTACAGACTCAGCCAATG | CTTTGCTCACTCTCTTGCCC |
|  | Exon 41 | TAGCTTTGAGGAGAGGTGGG | AATTCTCCACCAAGCCATTG |
|  |  | GACACTCCCAGGGGCAC | CAACACAGAGTCAATCCAGGG |
|  | Exon 42 | TGAACTTCAATTGCTCTATTCCTG | AAAAGCCTCCTTCATTTCCC |
|  | Exon 43 | AAAACGTTCATCTGAAACAAAAC | TGCTGACTGTTACAGCTGGG |
|  | Exon 44 | TAGAGGGGTGGAAGGAAATC | CAAAATGATGTGTACATGGGG |
|  | Exon 45 | CATTTCCAAAACAAAGGCTCTC | AAGCATTTTAGCCTCCACCC |
|  | Exon 46 | GAGGAACCCTTTGCTAAACC | CCACTTGAAGACATAGCCTGG |
|  | Exon 47 | AATGAGGGAAGGTGGGATTC | TGTCATGGCTGAGAGGATACC |
|  | Exon 48 | TCACGCCATGTGTTATTCTTC | AGCATCCTTTCTTTTCCGTG |
|  | Exon 49 | AGTAATATGGTCTCAGATCTTCTATGC | TGGTTCAATTTTTGTTGAGAGG |
|  | Exon 50 | TGGGATCTGCAAGATGATTG | GGTAGACCTGGGCCCCTTAC |
|  | Exon 51 | ATTTCAGCAACTGCCTGAGC | GAAAATGAATAATGCACACAGG |
|  | Exon 52 | AAAGTCACAAAAGCCTACCCTATG | GCAGCTTCTCACTAACCCC |
|  | Exon 53 | TGAAAATAAGAGAAACTTCCCTGTC | TGAATTTATGGGATTTTGTTATTTG |
|  | Exon 54 | TTAGGAACTGCTTGAGACAGC | CGTGACACATGAACACGCTAC |
|  | Exon 55 | ACATTCTCATTGATCAGGATAGC | TATCAAACACACACCCCTGG |
|  | Exon 56 | TTTTGCCCAATACTTAGCCTC | AAGCCTGAAGAATGGGAACC |
|  | Exon 57 | GGGGATGGTGTGACTTTTG | CAACTTTCAGTGGGACATGC |
|  | Exon 58 | GGCAAAGAGTTTGCAATTTGTC | TTTATCCAGGAGACCGACTATG |
|  | Exon 59 | CAAACATTTGTTGCCCATTC | TGCTTTACTCGATGCCAGAC |
|  | Exon 60 | ATGATATCATTACCCTTCTGTATG | ATGCTCTTGATTCTGCTGTGTTGG |
|  | Exon 61 | GTGTGCAGCTGTCACTGGTT | GCCCTAAGTGAAGAAAAATGAGA |
|  | Exon 62 | TACAAGCTTTGGCCATGAGG | TGTGAAGGGAGTTTTCCCAC |
|  | Exon 63 | AAAAGGGGCTAAGTA | TGTAGATTTTATATTCAC |
|  |  | AGGCCGACGAGAAAATTG | ATTTGAGTGGCACTGACG |
|  |  | CTATGCACTCCAAGCCTG | ACGATAGCGTGTTTCCAA |
|  |  | CCTGGAAACCTCCAAGAA | GGGCTCAGGCAATAGAAA |
|  | Exon 64 | AAGCTCTGCCTCCCAGG | ATGCAAATTCACCTGGAATC |
|  | Exon 65 | TTTTCCCAGCCTTAGGGTATC | AGAACAGAAGGATAAGAAAGATGG |
|  | Exon 66 | AATGCCATGTTGTGTCTCCC | TCAATAAGCAAGTCCTCCCTG |
|  | Exon 67 | CTGTGAAAACAAAATGTCTCCTC | GTGGCTTCTCCGAGTTTCAG |
|  | Exon 68 | GAACATACAACTTTCTCCCCACA | CGCCTTCCACACTGAGAAAC |
|  | Exon 69 | TGTGCCCTATTTGTAGGTGTTG | TGTGTGTTTCTTGAGGAAGATG |
|  | Exon 70 | GCAGTAAGAGACAAACTGCTTTC | CCTCTCTTGGTCCCCACAC |
|  | Exon 71 | TCCCTTCACTGACAATGTGTG | GCATTCGGTGAAGTACAGGC |
|  | Exon 72 | GCAAGGTCTGTTTTAGACCCCACC | ATGTGTGAGTGATAACCCAGGAGG |
| *ADGRV1* | Exon 1 | AGTACGGACGGGAGTCAGA | CAGCTATGCCCCATTACCC |
|  | Exon 2 | TGTCTTAGTTGTTTGCTGTCTAACTC | CCTTGCCATTAAAACTAAGAAACG |
|  | Exon 3 | CCTGGCCTGGAATCATTTG | GAAGCGCTGAAGAGGTTGTC |
|  | Exon 4 | TCCAGTACCTTCAATAGCATAGG | TGACCACGATCATACAAGCG |
|  | Exon 5 | TTTTACCGCCTCATACCTC | CTTGGGCAACAGAGCAAG |
|  | Exon 6 | GATGTATGGGTTAGTGTAATTCTTG | TCAGCTATTTATAGCTGAGGTGCG |
|  | Exon 7 | TTTATTTGCAGGTGTTTTGGC | TTCAGCAATCTCCGGTATGG |
|  |  | TCGTGGAAAGGACAACAATG | TTTGTCATGAAACTTTGTGGG |
|  | Exon 8 | CAACTTGACATTGGGAAAGC | TCTTTGGTTCTTGTACAGCACAG |
|  | Exon 9 | AAAATTCCACAAACTGTGA | TGACGGCAAAATAACAAGG |
|  | Exon 10 | TGTCACCCCATGCTGTTAAG | AAATGACATACTGAAGGCTTCTTG |
|  | Exon 11 | AATGCATTTAATAGTTCGCTTCTC | AAAAGTCTGAAGGCCAGTATAACAC |
|  | Exons 12&13 | CCTTAAAAGCCTGCAAAATTC | CCTCTTTCCTGTCTCTTTGCC |
|  | Exon 14 | CCTTTCAGTGAGTTATATGCTTCTG | TCTAAGGCAGGGTGAGCAAC |
|  | Exon 15 | TGCTAAGCAAATAGTTGGC | GGTTTATGGTCAGACACTG |
|  | Exon 16 | AAGTTCTGCCTCCGAAAAGG | AACTTCGAACAATTAAATAACAATCC |
|  | Exon 17 | GGGTAGTCATGTCAGAGTTGGG | TTCTTCACCCCATTTCTCAAG |
|  | Exon 18 | CCTTCAGTGAGGAAATTCT | TTTGAAGATAAGTGGCAAC |
|  | Exon 19 | TTTCCTTAATAACACAGGA | CCAATTGCATTTTGTATAT |
|  | Exon 20 | CCACATGGTGACACTACTTTTC | CATTCGTATTGGCATTGGG |
|  |  | TGGTTTGCCACCAATGATAG | TCTTGGTAGGCACATAGTTTTG |
|  | Exon 21 | TCACACGTAAAATTTAGGACAGG | GAGTCCCAAACAAATCCACC |
|  | Exon 22 | AGGATTTCATGGAAGCATTG | AAAGCTGCAAAGAAATTACAGAAC |
|  | Exon 23 | TGCAGAAATAAGTCGTAAGTGGTAAC | GGTCTGAAGGCATTCTACTTCC |
|  | Exon 24 | GGCCTGGTGATCAAAATAATTG | GGCGTGAGTCACTATGTTCC |
|  | Exon 25 | GGGATATAACAAACATTCTGATTTTAC | GAATCTACTTTCATCAGCATGGG |
|  | Exon 26 | TGCATTTTCCTCTTTGCTTG | GGTGTCAATAAGTGGTTAGTATTGTC |
|  | Exon 27 | CACCCATTTGTGTCTGCC | ATAGGCTCATTCCCCTCAGC |
|  | Exon 28 | GCCTCTAAGTAACTGGCTTTAATC | TGTTTCAGGATTCTGAGAGCTAA |
|  | Exon 29 | TTTGGCCAGTTCTTAGAA | AGGATACATGTCCCTCTG |
|  | Exon 30 | CCAAGTAGATCCATAAAGTTTGTTACC | GTCCTATATGTCAGTAACAGCAGG |
|  | Exon 31 | CCACTATAGGATGGTGCT | TCCTCTCCAAAATGTTAC |
|  | Exon 32 | TCTGTTACATGCTGGGTTTG | CAAGGGATCCTTCCCCAC |
|  | Exon 33 | AAGACCACAGTCAGCATTCCTC | AGAAAACTCTCATCCATCTCTGGG |
|  |  | GTGGCTGGGAGTGACTATGAGCC | TCATGTCAACCATTAACAAGCC |
|  | Exon 34 | TTGCCCATGATATGGAACTG | AAAGAACTAGACAAAAGACAACATCC |
|  | Exon 35 | TTGGGATTTGGGTTTTGTTG | TCCTCAAAATACACCATTCTTTC |
|  | Exon 36 | TTTGCCTGCACAATTTAT | TTGATCTAACATTTCTAG |
|  | Exon 37 | TTTCCAGCGATTAAAAGC | AATGGCTTTCATCCACAC |
|  | Exon 38 | TTTCCCACAGTGAGACAC | CATGAAACTTGATGTCTT |
|  | Exon 39 | TGGGTCAACCTAATTACT | AGCACAATGATCACTTCT |
|  | Exons 40&41 | AAAGGGACTGTCTTTAAATCCTATATC | TGGGACCTAAATCACTGTAATTCTC |
|  | Exon 42 | AACCAAAATATAAGGAGACAAAATTC | CCCATATTAAGGAACCCAGC |
|  | Exon 43 | TCACCTTAGGTAAAGAGTTGGC | TCTTTGTCTAACACCTAAGCTGG |
|  | Exon 44 | AAACAGATGTAACCATCACATTATAGG | AACTGGTCTCTCTAGACCTATCTTTTC |
|  | Exon 45 | TTCAGAATATAGAGTGCAAAGTTACTG | TTACAGGCATGAGCCTCAAC |
|  | Exons 46&47 | TCCCACTTGTGTCTTTTCACAC | CCCCGGATTAAGATACTTTGC |
|  | Exon 48 | TGCTATTAGTGTGTTCAGTGGC | AACCAGCAGACCATACCTTTG |
|  | Exon 49 | AGAGCAGCAAGGGAGCATC | ATCCATTTACAGCTGACAATTAATAC |
|  | Exon 50 | TTGGACTAAATGGATTCTGAGG | CCAGTGTCTGGCAATGTGATAC |
|  | Exon 51 | TTTTGGAAGATTTTGCTTTGG | CAAATTTCAGGCTGATAAATAGCTC |
|  | Exon 52 | AAATGTTGTGATTCTTCCAAACC | GGGAGAGAGGCTGATGACAC |
|  | Exon 53 | CATGTTGCTTAGGACCACAAAG | ATAAGGCAGGATTTGGTCCC |
|  | Exon 54 | AAAGTTTATAAATGGTTTGCTCTTCC | AAAGGATTGAGTATGACCTGCC |
|  | Exon 55 | ACCAAGCCCAGAATGATCC | ACTTAGGTGGGTGGCAACAG |
|  | Exon 56 | TCAAGTATTCAATGCAAGTTAAATGTC | CCAAATCAGCAGGGAGAGG |
|  | Exon 57 | AAGAGTTCAGAAGACCTTTTATGC | GAGTATAATGCATTTAGCAATCACC |
|  | Exon 58 | TCCTATCTTCCTCCTTCTTTTCC | TTTCTACTCAAAATTACATGAAACGC |
|  | Exon 59 | TTTTGTTTGGCCTTACTGAATTT | TCAAAGACAAATTGGGGAAAA |
|  | Exon 60 | CATTCAAACTTTGGCTTTGG | AATTATGAAACCTAACCCTGTCC |
|  | Exon 61 | GAACTAGATTCCTGTGTGTAAAGTGC | ATGCTTGAAGACTATGTATGTATTGAG |
|  | Exon 62 | TGAAATGGATGAAAATTGATTACTG | GCACACACCAGCACAGAGAG |
|  | Exon 63 | AAGAGTGGGAATGAGGAGGAG | AAAACTCTGGAATAGAAACAAGAAAAC |
|  | Exon 64 | TTATATGTAATTTTAACACAATCCTGG | CACAAATCTGAGAAAGAGTAGGG |
|  | Exon 65 | GCTAAGAGGATCTTTTAATTCATGC | CACACGGAAATTCAAACTAATG |
|  | Exon 66 | TGCCAGGTTTAATTTGGCTG | TTCACCTGTTGTTTTCCAATG |
|  | Exon 67 | GGAAAATGACTGGTTATTGGG | CTTGGGCAATGAGAAAACAC |
|  | Exon 68 | AACAGGAATTTGATGGAATTATCC | TTTATAATTTCCAATTTATCACAAGC |
|  | Exon 69 | GGGAATAAGAACCTACTTGTTTTAGG | TCCCTGTCCCTCTTAACCAC |
|  | Exon 70 | AGAGAGTTTGGCTTTGAATATAGTG | GTTACTAAACATTTTCGAGGGC |
|  | Exon 71 | TGCTAATGGCTCAAGTCAAAG | TTCCTGTAAAAGCTGCCCTC |
|  | Exon 72 | TTTACGTTTAACCCATTCCTCAG | TTAGTGGCCAGTGATATCCTTC |
|  | Exon 73 | CTACTACAGTTTTAAAAGTCAAATCCC | TGGATGAATTCCAGATTATTCCTC |
|  | Exon 74 | AATGATGAATCTCTACAGTCATATTCC | TACAGCCACAGTTGTCTCGG |
|  |  | AGCCTGTTCAGAATGGGG | TAAACCCACCAGTTCTTCGG |
|  |  | AACTTGTCACCCTTCATGGC | TGTCATGGGTCCATGCTAC |
|  | Exon 75 | TTAGTGGAGCATGGGAGGTC | CTGCCCTCCACCATTGTATG |
|  | Exon 76 | TGGATGAAGAGGTACCATTATTTG | AAAGAATCTCCCTATCAACAATGTG |
|  | Exon 77 | AAAGAAAGACCAAAAATAAATGTCA | TTCCCTATGTCATTACTCTTGCTG |
|  | Exon 78 | TTTGTTTAGGACAAGATCAGAGG | TGTGATTTTGGTTTTACTTGGG |
|  | Exon 79 | AATCTGTGCAGGCATTTAGG | CAACTTTCCAAGTGGTACAATGC |
|  | Exon 80 | CATGAAGAATAGAATCCAAGTGTAATG | AAATGCATTCCAAGTGTGCC |
|  | Exon 81 | CAAGTTCAGGGTAAGAGACTCAG | CTCAGTGGTACCGAAATGGG |
|  | Exon 82 | CCTGGACAAGATGTTCCCTTC | CCTCAGTACAGAGATGGCCC |
|  | Exon 83 | GACCTGACATGCCTAGCT | CCATTCTCTTCCACTGCA |
|  | Exon 84 | CAGTTTGGGCAAGAAGTCAAC | TCCTGAGAATGCAGTGATGG |
|  | Exon 85 | AAGCCAGTAGAGTTTGTG | GGCAACGAAATCTGCTTA |
|  | Exon 86 | GCATTTAGAAATATTTGCGCTG | AAAGATGAGCCGAACTTCCC |
|  | Exon 87 | GCACTAGAATACCACAGAAAGAAGC | TGTGTGTGGATGCTTGACTC |
|  | Exon 88 | CGGTAGCAGGACTGACTTTG | AGGTGGAGAGCCTGTCAATG |
|  | Exon 89 | TCAATTGTCCATTTGGGT | GGTGACTGATAGTACTTT |
|  | Exon 90 | AAACTGCCAAAGGAAATGTAAAAG | GCAGTGAATTTTCAGGTCACATAC |
| *DFNB31* | Exon 1 | CAGCAGCCAACTCTTGTGTC | GACCGGACTGTCCAGCAG |
|  |  | CTGCACCAAGCGCTGAC | AAGCTGTGCCACTCTGTCC |
|  | Exon 2 | TCTTCCATGTGGGGCTCCTGAG | TTCAGTACAGACTTGAGGGAAGAC |
|  | Exon 3 | TGCCAGTCGGATAAGATGCGCTC | AGCTGGGACCAGTCCTCAAGGTGGAG |
|  | Exon 4 | CTGGATGACCTCCTTGACAGGCTG | AGGCAGCTATCACTGGCTTTCAG |
|  | Exon 5 | ACTGAACTGGCAGCGAGTGGAGGC | TGCCCAGTTGGAATGAGGTAGTGTA |
|  | Exon 6 | AGATAGTACTATCAAGGCAAGGTG | AGGTCTCTGCCCAGTGTTCAGTTC |
|  | Exon 7 | AGCACTGGGGAGCCAGACAGTTAAG | TGCTGGGATTCGAACTCAGGCTGG |
|  | Exon 8 | AACCACATGGAGGTGTCCTAAGAC | ACCAGAGGCAAGTGATCCTGGTG |
|  | Exon 9 | AGCAGTGTGGCCAAGAGCAGTTAG | TCACTGTGTCATCAGGTAGACTGAC |
|  | Exon 10 | AGGCAGATGTCCTGGAAGTCACAG | GAGCTCACTACCTCCCTTTGTGGTC |
|  | Exon 11 | TAGGCAAGCTCCTTAACCTCCTGAG | TGACATAAGCCTAGGTCTGCCCTTG |
|  | Exon 12 | ACCCAGTTGGTGGTCAAAGCCAG | TGTCCTGCTCTCTTCCTCTCCCAG |
| *CLRN1* | Exon 1a | TCGATGGTGAAGTTGCCTTTTCAG | TAGGAAGAAGAAATTGCTCAGAGTC |
|  | Exon 1c | ACGAGTGAACGAGTGCAGGAACC | ACAAGAATTCAGGATGCACAAATTG |
|  | Exon 2 | AGTGAGAATGTATTCATCAGAAGG | TATTGAATTCCCTACTGTTGAGCAAG |
|  | Exon 3a | ATGGTAAGAGAATTCCTTGGTTTAC | TGTAAACATTGTCACGAAGGGTG |
|  | Exon 3c | TCTGGGATTATGACGGTATCACTG | TCAGAGGCCTAGTGATCTGTTTGC |

**Supplementary table S1: Primers used to amplify the exons of the USH genes**
